# Exosome to Promote Cancer Progression via Its Bioactive Cargoes

**Published:** 2021-06

**Authors:** Austin McMasters, Kelly M McMasters, Hongying Hao

**Affiliations:** The Hiram C. Polk, Jr., MD Department of Surgery, University of Louisville School of Medicine, Louisville, KY 40292, USA

**Keywords:** Exosome, Cancer progression, microRNA, circRNA, lncRNA

## Abstract

Exosomes are nanosized, organelle-like membranous vesicles secreted from various cell types, including normal cells and cancer cells. Exosomes contain abundant bioactive molecules, including nucleic acids, lipids, and proteins and dynamically participate in intercellular communications. By shuttling the functional molecules into the recipient cells, exosomes secreted by cancerous cells can alter the cellular environment to favor tumor growth and metastasis. In this review, we focus on exosomes to promote cancer progression via their various bioactive cargoes through different mechanisms/pathways. By recognizing these pathways, we can design efficient therapeutic strategies to control cancer progression.

Exosomes are membranous vesicles ranging in size from 30–100 nm in diameter. They are secreted from multiple cell types into the body fluids through exocytosis, a process commonly used for receptor discharge and intercellular communications [1]. The concept of exosomes has evolved since its inception. A more generalized term is extracellular vesicles (EVs), which are classified into exosomes and microvesicles (MVs) [2]. They are both double-layer phospholipid membranous vesicles, but differ in their size (exosomes: 30–100 nm in diameter; MVs: 100–1000 nm in diameter) and origin of cellular compartment [2]. The two types of vesicles have shared biological functions. We follow the term of exosome in this review. Exosomes contain abundant bioactive molecules, including nucleic acids, lipids, and proteins [3]. By shuttling the functional molecules into the recipient cells, exosomes dynamically participate in intercellular communications and are involved in both physiological and pathological processes in the body [4,5]. For example, exosomes secreted by healthy cells can transport homeostatic molecules such as tumor-suppressing proteins, transcriptional regulators, and various necessary genetic information. Exosomes released from immune effector cells are capable of inducing effective immune response to inhibit cancer growth [6,7]. On the other hand, cancer cell-derived exosomes can circulate within the body fluid and execute their biological functions through interorgan communication. Through this “long-distance” control mechanism, cancer cell-derived exosomes can prepare sentinel lymph nodes for cancer metastasis [8]. Exosomes released from cancer cells can trigger remote organ-specific pro-metastatic reaction to facilitate cancer metastasis through premetastatic niche formation at targeted organs [9].

Exosomes are observed at much higher levels in body fluids in pre-cancerous and cancer conditions than those in normal physiological conditions. Besides the change in quantity of the exosomes, the exosome components can also be altered in different biological conditions. One of the interesting finding is that diet can change exosome structure. High-fat diets can alter the lipid composition of exosomes from primarily phosphatidylethanolamine (PE) in exosomes from lean mice to phosphatidylcholine (PC) in exosomes from obese mice. After the intestinal exosomes from obese mice are taken up by macrophages and hepatocytes, they lead to the inhibition of the insulin signaling pathway and decreased insulin sensitivity [10]. However, whether or not diet can affect the cancer-cell derived exosome structure or secretion is not known yet.

In this review, we mainly focus on exosomes altering the cellular environment to favor cancer growth and metastasis through their active cargoes [3–5,11].

## RNA Cargoes in Exosomes

### MicroRNAs (miRNAs)

miRNAs are the most researched functional cargoes in exosomes. miRNAs are short RNAs (21–23 nucleotides) that bind to the 3’ untranslated regions of target genes, causing translational repression and rapid degradation of the target transcript [11,12]. Different miRNAs are involved in cancer progression through numerous pathways/mechanisms. Jiang et al. reported that, in breast cancer, the two miRNAs (miR-9 and miR-181a) derived from tumor exosomes can activate the JAK/STAT signaling pathway and promote the expansion of early-stage myeloid-derived suppressor cells (eMDSCs), thus cause immune escape and tumor growth [13]. Merkel cell carcinoma (MCC)-derived exosomes have a high level of miR-375 expression. The cancer cell-derived miR-375 acts as shuttle miRNA and is transferred into the fibroblasts. The fibroblasts go into polarization and change to the phenotype of cancer-associated fibroblasts (CAFs) through p53 pathway. This creates a pro-tumorigenic microenvironment favorable for cancer growth [14]. Exosome miRNAs also act as mediators between primary tumor cells and the distant organs. This communication is crucial for forming the pre-metastatic niche to promote tumor metastasis [15]. A review by Wortzel et al. discusses in detail of the roles of exosome miRNA and other exosome cargoes in the development of pre-metastatic niche and in organotropic metastasis [15].

With the recent progress of immunotherapy, a substantial amount of research has been focused on the role of tumor-derived exosomes in immune modulation to promote cancer progression [16]. Melanoma cell-derived exosomes contain miR-3187–3p, miR-498, and miR-149. Those miRNAs from exosomes can suppress CD8 T-cell cytotoxicity by regulating T cell receptor (TCR) signaling to promote cancer immune evasion [17]. miR-222–3p secreted from ovarian cancer exosomes can induce macrophage polarization from a tumor-suppressing M1 phenotype to a tumor-promoting M2 phenotype, therefore to facilitate disease progression [18].

Another emerging field is exosome miRNA-mediated metabolic reprograming to promote cancer progression [19]. Tumors are usually in hypoxia status due to their rapid oxygen consumption. Studies have shown that exosomes produced by hypoxic cancer cells are highly enriched in immunomodulatory proteins and chemokines including CSF-1, CCL2, and TGFβ [20]. Through transferring of let-7a miRNA, hypoxic cancer exosomes suppress the insulin-Akt-mTOR signaling pathway and evade host immunity to enhance cancer progression [20]. Another study shows that miR-155 and miR-210 secreted from human melanoma cells can remodel stromal cell metabolism and induce the formation of pre-metastatic niche to promote tumor metastasis [21].

### Long non-coding RNAs (LncRNAs)

LncRNAs is a type of RNA transcripts of more than 200 nucleotides with a limited or no protein-coding function. LncRNAs are involved in many diseases including cancer because they can modulate many biological processes including cell proliferation, differentiation, and cell death [22]. Liu et al. has shown that the expression level of exosomal lncRNA 01133 (LINC01133) is high in pancreatic ductal adenocarcinoma (PDAC) patients and is correlated with poor overall survival rate. LINC01133 promotes the proliferation, migration, invasion, and epithelial-to-mesenchymal transition (EMT) of pancreatic cancer cells through Wnt/β-catenin pathway. Exosomal LINC01133 plays an important role in pancreatic cancer progression [23]. Another lncRNA, CRNDE-h, is found to be abundant in colorectal cancer (CRC) exosomes. They can be transmitted to CD4+ T cells and contribute to the differentiation of CD4+ T cells into T helper 17 (Th17) cells to promote cancer progression [24]. Haderk et al. found that chronic lymphocytic leukemia (CLL)-derived exosomal RNA can promote expression of PD-L1 and adopt an immunosuppressive phenotype in CLL patients [25]. Noncoding RNA hY4 is a functional element of CLL-derived exosomes acting through TLR7 pathway [25]. In gastric cancer (GC), LINC01559 can be transmitted from mesenchymal stem cells (MSCs) to gastric cancer cells. LINC01559 accelerates GC progression by upregulating PGK1 and downregulating PTEN to activate the phosphatidylinositol 3-kinase/AKT serine/threonine kinase (PI3K/AKT) pathway [26].

### Circular RNAs (circRNAs)

circRNAs is a group of noncoding RNA with a circular structure in eukaryotes. circRNAs have tissue-specific and cell-specific expression patterns. circRNAs act as microRNA or protein inhibitors (‘sponges’). They execute important biological functions by regulating protein function or by being translated themselves [27,28]. Because of the covalently closed structure of these transcripts, it is challenging to detect and quantitate this type of RNA. It is even more difficult to characterize their function and define their roles in diseases. With the recent advances in high-throughput RNA sequencing and computational tools, it makes us possible to illustrate their function in cancer [28]. circRNAs are found to be enriched and stable in exosomes [29,30]. Exosomal circRNA_102481 is shown to be significantly up-regulated in non-small cell lung cancer (NSCLC) with EGFR-TKIs resistance. Expression of exosomal circRNA_102481 is associated with advanced TNM stage, increased odds of brain metastasis, and reduction of overall survival in NSCLC patients. Exosomal circRNA_ 102481 could contribute to EGFR-TKIs resistance via the microRNA-30a-5p/ROR1 axis in NSCLC [31]. Li, et al reported that exosomal circRNA_0044516 is significantly upregulated from prostate cancer patients. circRNA_0044516 plays an oncogenic role in prostate cancer to promote prostate cancer cell survival and metastasis [32]. Exosomal circRNA-100338 is also found to promote the metastasis of hepatocellular carcinoma by enhancing tumor invasiveness and angiogenesis [33].

## Protein Cargoes in Exosomes

### Alpha-enolase (ENO1)

ENO1, one of the three major enolases, is a key regulatory enzyme in glycolysis and is widely present in various cells and tissues [34]. Recent studies showed that ENO1 is upregulated in hepatocellular carcinoma (HCC) cells and has even higher expression in highly metastatic HCC cells as well as in exosomes [35]. ENO1 can be transferred between HCC cells via exosomes. Exosome-shuttled ENO1 can upregulate integrin α6β4 expression and activate the FAK/Src-p38MAPK pathway to promote HCC growth, metastasis, and disease progression [35].

### Soluble E-cadherin (sE-cad)

sE-cad is an 80-kDa protein that is highly expressed in the ascites of ovarian cancer patients. It is a potent inducer of angiogenesis [36]. There is evidence to show that plentiful of sE-cad is released in the form of exosomes. Exosomes with positive sE-cad heterodimerize with cadherin on endothelial cells and induce a sequential activation of β-catenin and NFκB signaling. Activation of both pathways activates the angiogenesis process in ovarian cancer, thus promoting cancer progression [36].

### c-Src

c-Src is a membrane-associated tyrosine kinase with important functions in the signaling transduction to control cell growth and migration [37]. Hikita et al. reported that c-Src is localized in the endosomal membrane. Once c-Src in the endosomal membrane is activated, it can be encapsulated in exosomes and promote exosome secretion. The secretion of exosomes can not only contribute to the maintenance of malignant phenotypes, but also transduce oncogenic signals to promote cancer progression [37].

### EphrinB1

One of the most interesting findings is that cancer exosome can cause tumor aggressiveness by inducing cancer innervation through exosome-packaged molecule, EphrinB1 [38]. Recent studies have shown that patients with heavily innervated cancers suffer from increased metastasis and dismal survival when compared to those with fewer innervated cancers [39,40]. EphrinB1 is a single pass transmembrane protein ligand that can bind and activate the Eph receptor, tyrosine kinases [41]. EphrinB1 acts as an axonal guidance molecule in the development of nervous system [42]. Exosome-packaged EphrinB1 works as an axonal guidance molecule to induce neurite outgrowth and to promote cancer innervation [38].

From the discussions above, we may notice that each type of cancer has its own specific genetic signature. Since exosomes are derived from their original cancer cells, it is rational to recognize that the exosomes from different cancer types have their specific genetic profile that distinguishes them from each other. For example, different types of cancers have unique exosomal RNAs that can differentiate themselves and be used as cancer biomarkers [43]. Proteomic analysis in exosomes also show that protein profiles differ in diverse cancer types and subtypes, as well as stages [44,45].

Table 1 summarizes the mechanisms/pathways involved in cancer progression by selected bioactive cargoes within the exosomes. We want to mention that, there are far more biological molecules and mechanims/pathways within exosomes contribute to this process. The goal of identifying the mechanisms of exosome-mediated cancer progression is to target those specific molecules, control cancer growth and metastasis, and eventually increase overall survival. Instead of targeting specific molecules for control cancer progression, a more general approach is to control exosome release and/or exosome uptake. For example, Rab27 controls exosome release. Rab27 is stabilized by interacting with KIBRA. Knockdown of KIBRA leads to decreased exosome secretion, so it follows that KIBRA can be used to regulate exosome secretion [46]. Other endocytosis inhibitors are used to block the uptake of exosomes as an alternative strategy to inhibit the malignant cell growth [47].

In summary, exosomes have been shown to promote cancer progression via their various bioactive cargoes. Much progress has been made on its mechanisms throughout the years. Accordingly, innovative strategies have been designed to target those molecules/pathways to control malignant progression. Since cancer and the surrounding microenvironment has a complex context and dynamic cross-talk, it is still challenging to design personalized medicine to control cancer progression.

## Figures and Tables

**Table 1:** Functions of bioactive cargoes in exosomes.

Bioactie cargoes		Names of specific cargoes	Cancer type	Pathways/Mechanisms	References
RNAs	miRNAs	miR-9 and miR-181a	Breast cancer	JAK/STAT signaling pathway	13
		miR-375	Merkel cell carcinoma	Fibroblasts polarization to cancer-associated fibroblasts/P53 pathway	14
		miR-3187–3p, miR-498, and miR-149	Melanoma	Cancer immune evasion	17
		miR-222–3p	Ovarian cancer	M2 macrophage polarization	18
		let-7a	Melanoma	Immune evasion/Insulin-Akt-mTOR signaling pathway	20
		miR-155 and miR-210	Melanoma	Remodel of stromal cell metabolism	21
	LncRNAs	LINC01133	Pancreatic cancer	Epithelial-to-mesenchymal transition/Wnt pathway	23
		CRNDE-h	Colorectal cancer	Immune suppression	24
		LINC01559	Gastric cancer	PI3K/AKT pathway	25,26
	circRNAs	circRNA_102481	Non-small cell lung cancer	EGFR-TKIs resistance	31
		circRNA_0044516	Prostate cancer	Oncogenic signaling	32
		circRNA-100338	Hepatocellular carcinoma	Angiogenesis	33
Protein		ENO1	Hepatocellular carcinoma	FAK/Src-p38MAPK pathway	35
		Soluble E-cadherin	Ovarian cancer	β-catenin and NFκB signaling	36
		c-Src	Colon cancer	Oncogenic signaling	37
		EphrinB1	Head and neck squamous cell carcinoma	Cancer innervation	38
